# Reovirus infection induces stabilization and up-regulation of cellular transcripts that encode regulators of TGF-β signaling

**DOI:** 10.1371/journal.pone.0204622

**Published:** 2018-09-27

**Authors:** Liang Guo, Jennifer A. Smith, Michelle Abelson, Irina Vlasova-St. Louis, Leslie A. Schiff, Paul R. Bohjanen

**Affiliations:** 1 Program in Infection and Immunity, Division of Infectious Diseases and International Medicine, Department of Medicine, University of Minnesota, Minneapolis, Minnesota, United States of America; 2 Institute for Molecular Virology Training Program, Graduate Program in Comparative and Molecular Bioscience, University of Minnesota, Minneapolis, Minnesota, United States of America; 3 Department of Microbiology and Immunology, University of Minnesota, Minneapolis, Minnesota, United States of America; Colorado State University, UNITED STATES

## Abstract

Reovirus infection induces dramatic changes in host mRNA expression. We utilized oligonucleotide microarrays to measure cellular mRNA decay rates in mock- or reovirus-infected murine L929 cells to determine if changes in host mRNA expression are a consequence of reovirus-induced alterations in cellular mRNA stability. Our analysis detected a subset of cellular transcripts that were coordinately induced and stabilized following infection with the reovirus isolates c87 and c8, strains that led to an inhibition of cellular translation, but not following infection with Dearing, a reovirus isolate that did not negatively impact cellular translation. The induced and stabilized transcripts encode multiple regulators of TGF- β signaling, including components of the Smad signaling network and apoptosis/survival pathways. The coordinate induction, through mRNA stabilization, of multiple genes that encode components of TGF-β signaling pathways represents a novel mechanism by which the host cell responds to reovirus infection.

## Introduction

Viral infection leads to changes in cellular steady state mRNA levels within infected cells. Some of these alterations represent the cell’s innate antiviral response, while others are induced by the invading virus in an attempt to sequester host antiviral responses and usurp the cellular machinery for viral replication. Virus-induced changes in cellular gene expression are often regulated through transcriptional mechanisms. For example, infection with many viruses increases the transcription of genes involved in antiviral responses including the type I interferons (IFN) as well as numerous IFN-stimulated genes (ISGs) (reviewed in [1, 2]). Although transcriptional regulation is important for mammalian cells to respond to their environment, numerous mammalian genes are also regulated at the level of mRNA decay in response to a variety of external signals (reviewed in [3, 4]. Virus-induced changes in cellular steady state mRNA levels have also been shown to be regulated at the level of mRNA decay [5, 6].

In this study, we utilized reovirus infection to evaluate the effect of viral infection on host cellular gene expression at the level of mRNA decay. Reovirus, a prototypic member of the *Reoviridae* family, is a non-enveloped double-stranded RNA virus that has been studied extensively as a model of viral infection [7]. This virus was a valuable model for us to examine the effect of viral infection on mRNA stability for several reasons: i) the consequences of reovirus infection are well documented and include induction of type I IFN, initiation of apoptosis, inhibition of cellular translation, and a G1/S cell cycle arrest (reviewed in [7]); ii) global studies on the impact of reovirus infection on cellular gene expression have been published [8–11]; and iii) reovirus isolates vary in their effects on infected cells [10, 12–14]. Since reovirus isolates induce distinct changes in cellular gene expression, as well as distinct cellular responses to infection, alterations in cellular gene expression following reovirus infection can be correlated to specific phenotypes [10]. For example, in murine L929 cells, reovirus isolates Dearing and c87 induce high levels of type I IFN, whereas cells infected with isolate c8 have a poor IFN response [10]. As a consequence, numerous ISG transcripts are induced following infection with isolates Dearing or c87, but not isolate c8 [10]. Additionally, infection with c87 or c8 lead to an inhibition of cellular translation, whereas infection with Dearing does not [10, 12, 14]. Mechanisms for inhibition of cellular translation in response to reovirus infection involve phosphorylation and inactivation of the alpha subunit of eukaryotic initiation factor-2 (eIF2α) by the dsRNA-dependent protein kinase (PKR) or the ER-stress-induced kinase PERK [10]. We previously identified a specific subset of cellular transcripts that were induced following infection with c8 and c87, which inhibit cellular translation, but were not induced following infection with isolate Dearing, which does not inhibit cellular protein synthesis [10]. The mechanisms for increased steady state expression of these cellular transcripts could involve transcriptional and/or posttranscriptional mechanisms.

In this report, we investigate the role that mRNA decay plays in regulating host cellular gene expression following reovirus infection. We used oligonucleotide microarrays to measure mRNA decay rates in L929 cells that were mock-infected or infected with reovirus isolates Dearing, c8, or c87. We detected a subset of transcripts that were coordinately induced and stabilized upon infection with reovirus strains that induced host translational shutoff, i.e. strains c8 and c87. The induced and stabilized transcripts encoded multiple regulators of transforming growth factor-beta (TGF-β) signaling, including components of the Smad signaling network (SSN) and apoptosis/survival pathways. TGF-β is a cytokine that has multiple activities including immune modulation, promotion of fibrosis, control of cellular growth, and regulation of apoptosis [15–20]. TGF-β production is activated following infection with a variety of viruses [21–28], including reovirus [29, 30], suggesting that the TGF-β signaling cascade plays a role in viral pathogenesis. In particular, regulation of apoptosis through TGF-β signaling may be part of a host response to viral infection. Thus, the coordinate stabilization and up-regulation of transcripts that encode components of TGF-β signaling pathways likely represent a cellular anti-viral response to reovirus infection.

## Methods and materials

### Cells, viruses and viral infection

Murine L929 cells were maintained as suspension cultures as described previously [31]. Reovirus isolates Dearing and c87/Abney are prototypic laboratory strains [32], and isolate c8 was previously described [31, 33]. Purified virions were prepared by CsCl density gradient centrifugation of extracts from cells infected with third-passage L929 cell lysate stocks [34]. In order to analyze three independent infections, each set of infections was initiated on separate days. L929 cells were plated in 150 x 25 mm tissue culture plates and were allowed to incubate at 37°C for 4 h, after which time the medium was removed and cells were mock-infected or infected with purified virions (Dearing, c87 or c8) at a multiplicity of infection (MOI) of 80 plaque forming units (PFU)/cell. After a 1.5 h viral adsorption at 37°C, medium was added and samples were incubated at 37°C for an additional 18 h.

### RNA isolation and microarray hybridization

At 19.5 h post-infection (p.i.), actinomycin D (Sigma, MO) was added at a final concentration of 10 μg/ml to stop transcription by RNA polymerase II and total RNA was isolated at 0, 45, 90 and 120 min post-actinomycin D treatment using Trizol reagent (Invitrogen, CA) according to the manufacturer’s instructions. Total RNA was purified with the RNeasy column (QIAGEN, CA); 15 μg of RNA was converted to cDNA using the Superscript custom kit (Invitrogen) with an oligo dT-T7 primer (Geneset, CO). The purified cDNA was used for an *in vitro* transcription reaction using T7 RNA polymerase and biotinylated nucleotides following the manufacturer’s protocol (ENZO Bioarray, NY). Biotinylated anti-sense cRNA was purified with the RNeasy column; 15 μg was fragmented according to Affymetrix instructions’ and hybridized to Affymetrix murine U74Av2 oligonucleotide microarrays (Affymetrix Inc., CA). Microarrays were scanned on a Hewlett Packard Agilent 2200 confocal scanner (Bio-Rad Laboratories, CA) and normalized signal intensities were obtained using Affymetrix MAS 5.0 software as described previously [10].

### Microarray data analysis

Expressed transcripts levels were determined as the average signal values with 95% confidence intervals (95% CI) across three replicate arrays. Fold changes (FC) in expression between two infection conditions were determined as the ratio of average signal values. P values were calculated using a two-sample t test assuming equal variance. We fit the log signal values over time following actinomycin D treatment to a linear regression model over the linear portion of the decay curves to calculate transcript half-lives as described in detail in a previous statistical supplement [35]. A p-value of ≤ 0.05 was used to identify differences in mRNA decay rates in mock-infected cells compared to reovirus-infected cells.

### Reverse transcription real-time PCR

Reverse transcription real time PCR (RT-PCR) was used to validate changes in transcript level and mRNA decay rates of three transcripts that were found to be up-regulated and stabilized following infection with certain reovirus isolates: Gdf15, Tgif, and Myc. Total cellular RNA from the same reovirus infections as described above was used for real time RT-PCR. RNA was converted to cDNA by using StrataScript^™^ reverse transcriptase (Stratagene) and gene specific primers. PCR amplifications were performed in a BioRad iCycler thermocycler by using the QuantiTect^™^ SYBR Green PCR Kit (QIAGEN) with the following cycling conditions: initial heating at 95°C for 13.5 min, followed by 40 cycles of 3-step temperature cycling at 95 °C for 10 s, 55.6 °C for 10 s, and 72 °C for 30 s. Data was analyzed using the iCycler software and standard curves were generated to measure transcripts levels, which were normalized to the level of HPRT transcript. The normalized values at each point were then used to generate mRNA decay curves. Oligonucleotide primers (Integrated DNA Technologies Inc) were: Gdf15 5’ CCG AGA GGA CTC GAA CTC AG 3’, 5’ GTA GGC TTC GGG GAG ACC 3’; Hprt 5’ GGT GAA AAG GAC CTC TCG AA 3’, 5’ AGT CAA GGG CAT ATC CAA CA 3’; c-Myc 5’ TGA AGG CTG GAT TTC CTT TG 3’, 5’ TTC TCT TCC TCG TCG CAG AT 3’; Tgif 5’ TCC TAG AAA CCC CAG CTT CA 3’, 5’ GCT GCT GAT GAG GAA AGG TC 3’.

## Results

We profiled the changes in L929 cellular gene expression and mRNA decay rates that occurred as a consequence of infection with reovirus isolates c87, c8 and Dearing. At 19.5 h p.i., a time point where reovirus-induced host translational shutoff is well established [10], ongoing transcription was arrested by addition of actinomycin D and global mRNA expression levels were measured after 0, 45, 90 and 120 min of actinomycin D treatment using Affymetrix U74Av2 microarrays as described previously [35, 36]. This experiment was performed three separate times for each reovirus isolate and the mRNA half-life with 95% confidence interval (95% CI) was calculated for over 6500 expressed transcripts based on a model of first order decay. The transcript levels and mRNA decay rates for all transcripts expressed under at least one condition are shown in S1 Table and the complete set of raw data has been deposited into the NCBI GEO Database (accession # GSE119061). The numbers of transcripts that were up-regulated and/or stabilized in reovirus-infected cells compared to mock-infected cells are shown in Table 1.

**Table 1:** Number of cellular transcripts that were stabilized and up-regulated following reovirus infection.

	Strain c87	Strain c8	Strain Dearing	Strains c87 and c8	All 3 Strains
Up-regulated (p<0.05)	709	481	590	305	205
Up-regulated (FC > 2-fold, p<0.05)	347	200	210	143	88
Stabilized (p < 0.05)	349	253	52	172	24
Stabilized and Up-regulated (p < 0.05)	70	49	2	26	0
Stabilized and Up-regulated (FC > 2-fold, p < 0.05)	31	41	0	13	0

We observed the stabilization (p ≤ 0.05) of 349, 253, and 51 cellular transcripts after infection with reovirus isolates c87, c8, and Dearing, respectively. A complete list of the stabilized transcripts and their decay rates are displayed in S2 Table. Of the stabilized transcripts, 172 were stabilized following infection with both c87 and c8 isolates, whereas only 24 transcripts were stabilized after infection with all three isolates. We also noted the destabilization (p ≤ 0.05) of a small number of transcripts in L929 cells following infection with these same reovirus isolates, but there was poor correlation between isolates; only four transcripts were destabilized following infection with both c87 and c8 and no destabilized transcripts were common to all three isolates.

We evaluated the steady state mRNA levels of stabilized cellular transcripts to determine whether or not stabilization following reovirus infection correlated with changes in overall levels of these transcripts. Following c87 infection, 70 cellular transcripts were up-regulated (p ≤ 0.05) and stabilized (p ≤ 0.05); 40 transcripts were up-regulated and stabilized following c8 infection (Table 1). Of these, 26 transcripts were up-regulated and stabilized following infection with both of these reovirus isolates. In contrast, only two transcripts were up-regulated and stabilized following infection with Dearing, the reovirus isolate that did not inhibit cellular translation. Using more strict criteria to identify transcripts that were up-regulated (FC ≥ 2.0 and p ≤ 0.05) in reovirus-infected cells, we identified 31 transcripts that were up-regulated and stabilized following c87 infection and 41 transcripts that were up-regulated and stabilized following c8 infection; 13 were up-regulated and stabilized following infection with both isolates that led to an inhibition of host protein synthesis. We did not identify any transcripts that were up-regulated ≥ 2-fold and stabilized following infection with strain Dearing.

**Fig 1:**
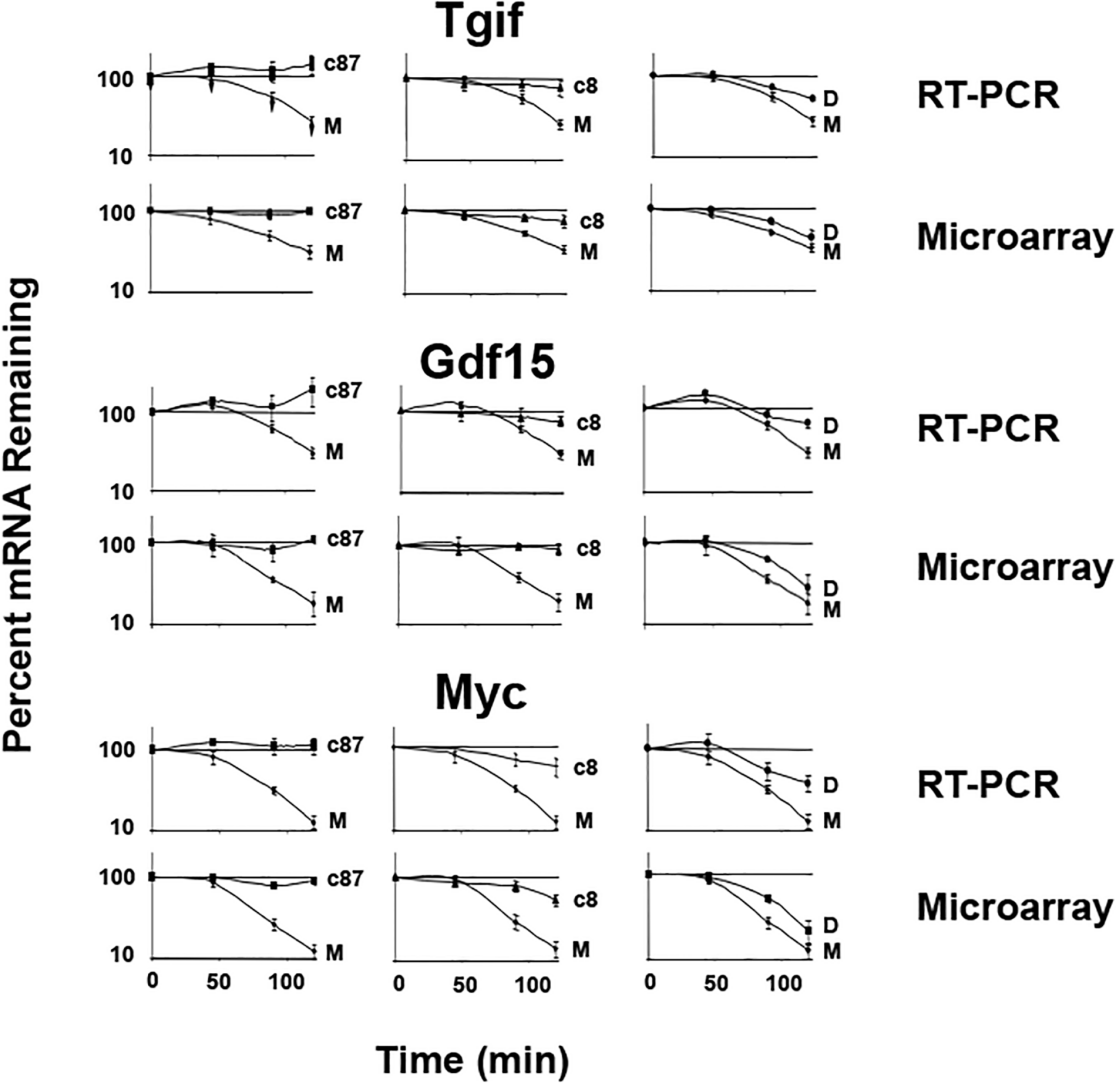
Real time RT-PCR validation of transcript up-regulation and stabilization. Murine L929 cells were infected for 19.5 h with reovirus isolates c87, c8 and Dearing or mock (M) infected. Actinomycin D was added to stop transcription and total cellular RNA was purified 0, 45, 90, and 120 min post-actinomycin D treatment. The same RNA was used for both microarry and real time RT-PCR. Tgif, Gdf-15 and c-Myc mRNA levels were measured by real-time RT-PCR using gene specific primers and transcript levels were normalized to the level of the HPRT transcript. Data shown are from three independent experiments. Each point represents the mean ± standard error of the mean.

A subset of the transcripts that were up-regulated (p ≤ 0.05) and/or stabilized (p ≤ 0.05) following infection of L929 cells with c87 or c8 are listed in Table 2. A complete list is found in S3 Table. These transcripts encode various components of the TGF-β signaling pathway (Fig 1), including cell cycle inhibitors and regulators of transcription, apoptosis and stress responses. In particular, many encode protein components of the SSN, which regulates transcription associated with cell cycle arrest, differentiation and apoptosis [37–41]. This suggests that the SSN is involved in a coordinated cellular response to infection with reovirus isolates c8 and c87. In contrast, none of the transcripts encoding components of this pathway were up-regulated and/or stabilized following infection with isolate Dearing, suggesting that reovirus isolates differ in their ability to induce changes in the decay of cellular transcripts encoding SSN components. The finding that strains that induced stabilization of transcripts encoding components of the SSN network also induced cellular translational inhibition suggests that the SSN network and inhibition of translation could be linked.

**Table 2:** Subset of transcripts that were stabilized or up-regulated following reovirus infection.

Transcript Description	Gene Symbol	Mock		Strain c87			Strain c8		
		HL(min)	95% CI	FC	HL(min)	95% CI	FC	HL(min)	95% CI
**Up-regulated (p** ≤ **0.05) and Stabilized (p** ≤ **0.05); c87 and c8**									
*growth differentiation factor 15	Gdf15	69	[47,124]	18.24	>480	[101,>480]	12.07	>480	[115,>480]
*MAD homolog 7 (Drosophila)	Smad7	67	[52,95]	4.03	197	[89,>480]	1.87	199	[90,>480]
*dual specificity phosphatase 1	Dusp1	50	[39,68]	3.65	>480	[112,>480]	3.65	104	[57,>480]
*myelocytomatosis oncogene	Myc	44	[37,55]	2.59	366	[108,>480]	2.20	183	[83,>480]
*vascular endothelial growth factor A	Vegfa	119	[79,240]	2.44	>480	[226,>480]	3.49	>480	[156,>480]
*TG interacting factor	Tgif	94	[76,124]	2.33	>480	[217,>480]	2.59	468	[174,>480]
*Kruppel-like factor 5	Klf5	115	[82,192]	2.18	>480	[149,>480]	1.78	>480	[179,>480]
coagulation factor III	F3	77	[55,130]	7.79	>480	[115,>480]	5.16	>480	[128,>480]
nuclear receptor subfamily 1, group D, member 1	Nr1d1	125	[89,207]	4.97	>480	[294,>480]	3.79	>480	[174,>480]
nuclear factor, interleukin 3, regulated	Nfil3	65	[49,100]	2.77	171	[75,>480]	2.85	184	[77,>480]
B-cell translocation gene 1, anti-proliferative	Btg1	152	[109,250]	1.68	>480	[398,>480]	2.15	>480	[209,>480]
CDC like kinase 4	Clk4	78	[59,115]	3.29	>480	[128,>480]	2.99	>480	[174,>480]
**Up-regulated (p** ≤ **0.05) and Stabilized (p** ≤ **0.05); c87**									
*dual specificity phosphatase 2	Dusp2	91	[60,180]	3.31	>480	[120,>480]	1.57	371	[96,>480]
*growth arrest and DNA-damage-inducible 45 beta	Gadd45b	53	[40,79]	2.18	139	[63,>480]	3.21	80	[48,259]
*MAD homolog 2 (Drosophila)	Smad2	216	[153,368]	1.28	>480	[286,>480]	1.15	>480	[335,>480]
immediate early response 3	Ier3	49	[37,71]	4.22	230	[75,>480]	2.17	113	[56,>480]
nucleoporin 62	Nup62	146	[101,264]	1.73	>480	[196,>480]	1.06	188	[104,>480]
seven in absentia 2	Siah2	72	[56,100]	1.78	>480	[174,>480]	1.29	454	[129,>480]
**Up-regulated (p** ≤ **0.05) and Stabilized (p** ≤ **0.05); c8**									
*signal transducing adaptor molecule 1	Stam	222	[142,>480]	1.45	>480	[341,>480]	1.81	>480	[340,>480]
*MAD homolog 1 (Drosophila)	Smad1	163	[123,242]	1.16	>480	[>480,>480]	1.36	>480	[252,>480]
B-cell leukemia/lymphoma 6	Bcl6	49	[41,61]	1.01	>480	[130,>480]	1.86	273	[105,>480]
cyclin G2	Ccng2	93	[71,134]	0.67	>480	[215,>480]	2.41	>480	[229,>480]
TGFB inducible early growth response	Tieg / Klf10	61	[51,75]	0.69	287	[126,>480]	1.49	145	[88,397]
E4F transcription factor 1	E4f1	373	[216,>480]	1.24	>480	[443,>480]	1.58	>480	[>480,>480]
**Stabilized (p** ≤ **0.05); c87 and c8**									
*mitogen activated protein kinase kinase kinase 1	Map3k1	77	[59,108]	0.48	>480	[157,>480]	1.27	399	[128,>480]
*Cbp/p300-interacting transactivator, CITED2	Cited2	71	[56,97]	0.69	>480	[138,>480]	1.47	264	[110,>480]
*BCL2-like 11	Bcl2l11	67	[52,94]	0.86	348	[112,>480]	0.79	261	[102,>480]
*Son of sevenless homolog 2, (Drosophila)	Sos2	93	[63,174]	0.97	>480	[120,>480]	1.57	>480	[121,>480]
*B-cell leukemia/lymphoma 10	Bcl10	374	[239,>480]	1.10	>480	[>480,>480]	1.18	>480	[455,>480]
*TANK-binding kinase 1	Tbk1	121	[85,210]	1.04	>480	[142,>480]	1.20	>480	[170,>480]

**Fig 2:**
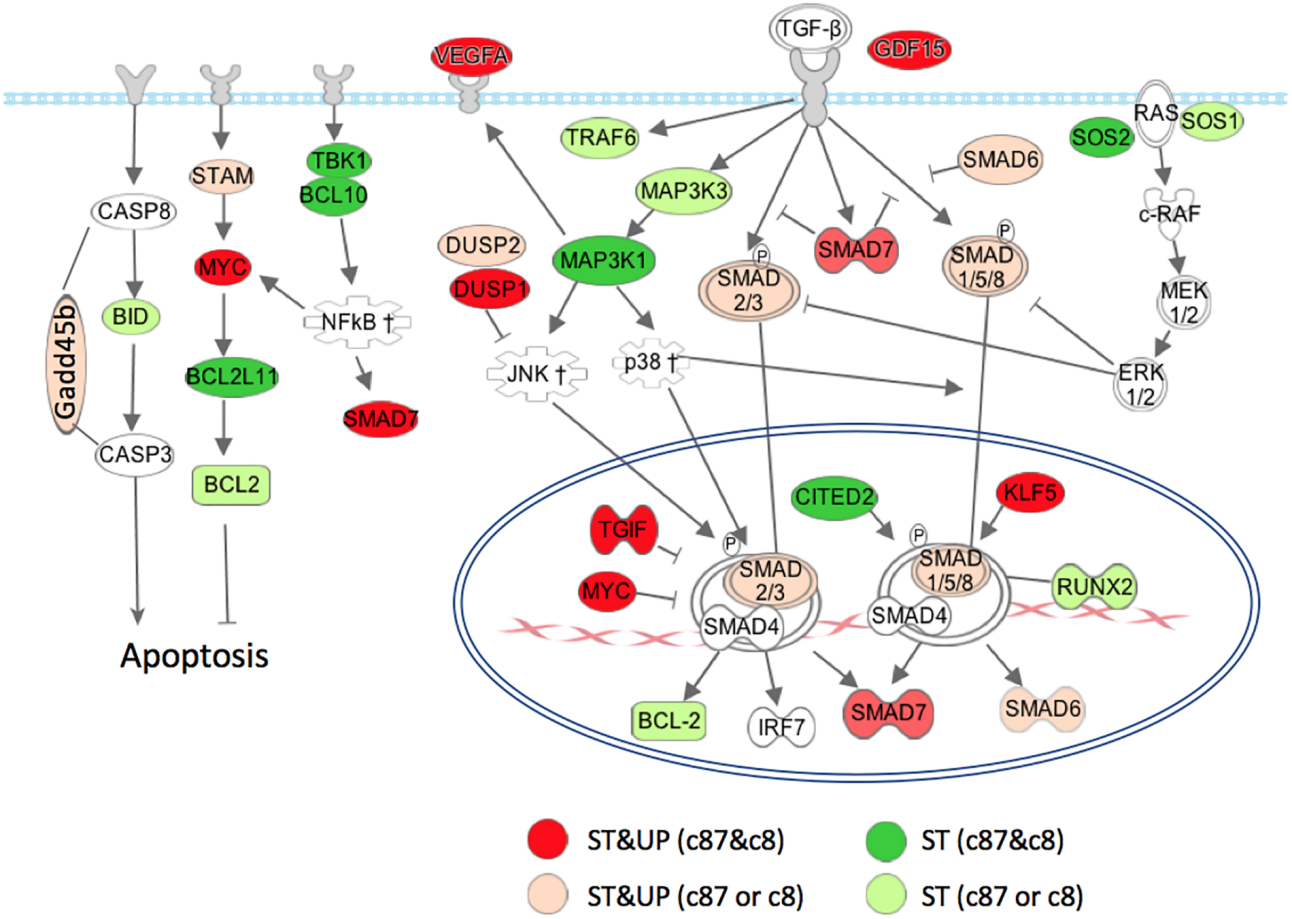
Transcripts that encode components of the SSN or related proteins were up-regulated and/or stabilized following reovirus infection. Signaling through the TGF-β family of receptors activate several pathways, including NF-κB, MAPK/ERK, p38, and JNK pathways. These pathways regulate phosphorylation of Smad proteins, which in turn regulate cell survival and apoptosis. Transcripts shown in red were up-regulated and stabilized following infection of L929 cells with reovirus isolates c87 and c8, transcripts shown in light orange were up-regulated and stabilized following infection with isolate c87 or c8, transcripts shown in dark green were stabilized (but not up-regulated) following infection with isolates c87 and c8, and transcripts shown in light green were stabilized (but not up-regulated) following infection with isolate c87 or c8. This figure was created using Ingenuity Pathway Analysis software starting with the canonical pathway related to TGF-β signaling (right side of the figure), which was combined with transcripts related to apoptosis (left side of the figure). GDF15, VEGFA, DUSP1/2, KLF5, and CITED2 transcripts were added manually based on their relevance to TGF-β or apoptosis signaling pathways.

*Transcripts shown in Fig 2.

To validate our microarray mRNA decay data, we utilized real time RT-PCR to measure mRNA levels from three selected genes: Tgif, Gdf15 and c-Myc. These transcripts encode important proteins that interface with the SSN and, based upon our microarray data, were up-regulated and stabilized following infection with c87 and c8, but not Dearing. We generated decay curves for the three transcripts by real time RT-PCR using the same RNA that was used for the microarray analysis. Decay curves generated for these transcripts by real time RT-PCR were very similar to the mRNA decay curves generated from the microarray analysis (Fig 1). These data confirm that Tgif, Gdf-15, and c-Myc were up-regulated and stabilized following infection with reovirus isolates c87 and c8 (Table 3). In contrast, infection with strain Dearing led to little or no stabilization of these transcripts (Fig 1 and Table 3). We also performed western blotting using antibodies against the Tgif, Gdf-15 and c-Myc proteins and found that the level of these proteins did not increase followinginfection with reovirus isolate c87 (S1 Fig). Thus, the upregulation and stabilization of these transcripts did not correlate with increased protein levels. This result is not surprising, as infection with this reovirus isolate led to an inhibition of cellular protein synthesis.

**Table 3:** Comparison of transcript expression and half-life data obtained using real time RT-PCR or microarrays.

Transcript	Mock		Strain c87			Strain c8			Strain Dearing		
	HL(min)	95% CI	FC	HL(min)	95% CI	FC	HL(min)	95% CI	FC	HL(min)	95% CI
**Tgif**											
RT-PCR	83	[71,103]	3.49	>480	[>480]	3.69	>480	[>480]	2.78	171	[147,195]
Microarray	94	[76,124]	2.33	>480	[217,>480]	2.59	468	[174,>480]	1.55	113	[80,191]
**Gdf15**											
RT-PCR	124	[85,174]	20.45	>480	[>480]	18.24	>480	[458,>480]	14.19	226	[151,>480]
Microarray	69	[47,124]	18.24	>480	[101,>480]	12.07	>480	[115,>480]	3.83	63	[40,149]
**Myc**											
RT-PCR	54	[47,61]	1.43	>480	[>480]	2.60	339	[223,443]	1.85	86	[75,98]
Microarray	44	[37,55]	2.59	366	[108,>480]	2.20	183	[83,>480]	1.89	56	[41,88]

## Discussion

We found that multiple cellular transcripts encoding components of the SSN are coordinately up-regulated and stabilized following reovirus infection. We hypothesize this is a cellular response to reovirus infection meant to induce the apoptosis of infected cells, particularly following infection with reovirus isolates that lead to the inhibition of cellular translation. Numerous viruses, including reovirus, induce increased expression of TGF-β as part of the host response to viral infection. Depending upon the integration of signals through the TGF-β receptor and other receptors, the SSN regulates the balance between apoptosis or cell growth and survival (reviewed in [42]). In virus-infected cells, TGF-β signaling may play an antiviral role by promoting the apoptosis of virus-infected cells, whereas in uninfected cells, signaling through the SSN may play a role in protecting against apoptosis [29]. In addition to activating the SSN, the TGF-β family of receptors interfaces with several other signaling pathways, including NF-κB, MAPK/ERK, p38 and JNK pathways [43–46]. These pathways influence the SSN by regulating the phosphorylation of Smad proteins, which in turn, control cell proliferation, differentiation and migration through their role as transcription factors [39, 41, 47–49]. Smad transcription complexes are activated by TGF-β receptors 1 and 2. Following receptor activation, Smad 2,3 complexes or Smad 1,5,8 complexes become phosphorylated and interact with Smad 4, creating activated transcription complexes [38, 50]. These newly formed Smad 4-containing complexes translocate into the nucleus, bind DNA, and activate target gene transcription. Depending on other signals, however, Smad 6 and Smad 7 can repress phosphorylation and prevent activation of Smad transcription complexes by blocking their translocation into the nucleus [39, 51, 52]. Smad 7 can also act in a feedback loop to repress TGF-β signaling by inducing receptor ubiquitylation and protein degradation [53–55]. Thus, depending on the integration of multiple signals, the SSN can activate or repress transcription of a specific subset of cellular genes.

Transcripts that were up-regulated and stabilized following reovirus infection included SMAD 1, 2, 6 &7, Tgif, c-Myc, CITED2 and KLF5, which encode components of the SSN that control transcription of genes that regulate apoptosis and cell growth [47, 50, 51, 53, 54]. Of note, reovirus has been shown to preferentially infect and induce lysis of cells that express high levels of c-Myc or other oncoproteins, suggesting that reovirus might exploit this oncogenesis signaling pathways to preferentially kill cancer cells [56]. For this reason, reovirus infection is a potential treatment of cancer [57]. Other up-regulated and stabilized transcripts encode growth regulatory cytokines that impact the SSN (see Fig 2). For example, the transcripts encoding Gdf15, a TGF-β superfamily cytokine, and Vegfa, an angiogenic endothelial cell growth factor, were dramatically stabilized and up-regulated following infection with reovirus isolates c8 and c87. Interestingly, Vegfa is induced by TGF-β and acts in concert with TGF-β to induce the apoptosis of endothelial cells [58, 59]. In addition to growth factors, transcripts encoding downstream regulators of kinase pathways that can also impact the SSN, including Sos1, Sos2, Map3k3, Map3k1, Dusp1, and Dusp2, were also up-regulated and/or stabilized. The transcript encoding Stam, a cytokine signaling protein that interfaces with the SSN by activating c-Myc [60], was also up-regulated and stabilized in cells infected with reovirus isolates c87 or c8.

Since the SSN regulates the balance between apoptosis and survival, it is possible that the coordinate induction of SSN components through mRNA stabilization represents an attempt by the virus-infected cell to undergo apoptosis. Some up-regulated and stabilized transcripts encode components of receptor-mediated apoptosis pathways including: Bcl-10, which activates NF-κB [61]; Tbk1, which promotes anti-viral responses [62] and activates NF-κB [63]; Gadd45b, a NF-κB-inducible mediator of apoptosis [64]; and Bid, an important component of caspase-induced apoptosis [65]. TGF-β signaling also leads to activation of NF-κB and promotion of apoptosis through the signaling protein TGF-β activating kinase 1 (Tak1) [45, 66, 67]. The NF-κB signaling network interfaces with the SSN by activating Smad 7, which feeds back to repress TGF-β-induced transcription of genes that promote cell growth and survival [45, 68]. Thus, the outcome of the SSN—cell growth and survival versus growth inhibition and cell death—is controlled by the coordinate integration of several signals. Following reovirus infection, not all cellular anti-viral responses lead to apoptosis; rather, a balance between death and survival occurs [69, 70]. Cells attempt to avoid viral infection, but if unsuccessful, cell death pathways are frequently activated. Meanwhile, viruses need to prevent cell death for a period of time to ensure viral replication. Thus, the interplay between host anti-viral responses to promote death of infected cells and viral evasion mechanisms determines the fate of the cell.

We have previously demonstrated that reovirus isolates that led to an inhibition of cellular translation (c87 and c8) also induced stress granule formation [8]. Others have shown that reovirus particles are recruited to stress granules during infection, and the stress response induced by reovirus may be necessary for viral replication [71]. Here we demonstrate that infection with the reovirus isolates that induced stress granule formation also caused the stabilization of numerous cellular transcripts, including transcripts encoding components of the SSN and regulators of apoptosis. Perhaps, stress granule formation leads to the stabilization and sequestration of certain cellular transcripts, such as the transcripts that encode the regulators of the SSN and apoptosis pathways we identified here. Thus, the stabilization and up-regulation of transcripts that encode components of the SSN and associated apoptosis pathways may be part of a cellular stress response in which these transcripts are stabilized within stress granules while the cell determines its fate (cell death or survival).

Numerous other viruses have developed mechanisms to modulate or usurp TGF-β signaling pathways, perhaps to prevent cell death and promote viral replication. For example, Kaposi sarcoma herpes virus produces viral homologues of human interferon response factors that function to regulate TGF-β signaling [72, 73]. Herpes simplex virus 1 down-regulates TGF-β and Smad 3 expression in infected cells [74, 75]. Although this effect was reported to be due to an HSV-1-encoded microRNA [74], other groups were unable to reproduce those results [76]. Human papillomavirus E6 and E7 proteins bind to specific Smad proteins, thereby inhibiting the SSN [77, 78], and the human T cell lymphotropic virus 1 tax protein inhibits TGF-β signaling through c-jun activation [79]. Other viruses, such as cytomegalovirus and BK virus, usurp TGF-β signaling to promote viral replication [80, 81]. The fact that numerous viruses have developed specific mechanisms to manipulate or evade the SSN suggests this pathway is important for host anti-viral responses.

Our findings demonstrate that transcripts encoding numerous components of the SSN are coordinately up-regulated and stabilized following reovirus infection, suggesting that cells have mechanisms to selectively recognize and stabilize specific subsets of cellular transcripts. These transcripts may contain specific regulatory sequence(s) in common that allow them to be selectively recognized by RNA-binding proteins or microRNAs. The finding that after reovirus infection, only a specific subset of transcripts undergo stabilization, rather than all transcripts, suggests that alterations in general pathways for mRNA decay cannot explain our results. It is possible that certain transcripts that are targets for translation-dependent mRNA decay under normal conditions are stabilized when translation is inhibited following reovirus infection. Nonsense-mediated mRNA decay, which is translation-dependent [82, 83], has been shown to regulate the decay of transcripts involved in the TGF-β signaling pathway [84, 85]. For example, many of the transcripts involved in TGF-β signaling depicted in Fig 1 have been shown to be targeted by nonsense-mediated decay, such as Smad7 [85], DUSP1/3 [86], GADD45B [87], and Myc [88]. Further work is needed to define the mechanism for the up-regulation and stabilization following reovirus infection of the transcripts we identified which encode specific components of the SSN.

## Supporting information

S1 Table Expression intensity and half-lives of all transcripts in mock, c87, c8, or Dearing infected cells. (XLS)

S2 Table Transcripts that were stabilized following reovirus infection. (XLS)

S3 Table Transcripts that were up-regulated and stabilized following reovirus infection. (XLS)

S1 Fig Western blot analysis of total cell lysates from mock and reovirus-infected L929 cells. (PDF)

## References

[1] Sadler AJ, Williams BR. Interferon-inducible antiviral effectors. Nat Rev Immunol. 2008;8(7):559–68. 10.1038/nri2314 18575461 PMC2522268

[2] Schoggins JW, Rice CM. Interferon-stimulated genes and their antiviral effector functions. Curr Opin Virol. 2011;1(6):519–25. 10.1016/j.coviro.2011.10.008 22328912 PMC3274382

[3] Schoenberg DR, Maquat LE. Regulation of cytoplasmic mRNA decay. Nat Rev Genet. 2012;13(4):246–59. 10.1038/nrg3160 22392217 PMC3351101

[4] Wu X, Brewer G. The regulation of mRNA stability in mammalian cells: 2.0. Gene. 2012;500(1):10–21. 10.1016/j.gene.2012.03.021 22452843 PMC3340483

[5] Guo L, Sharma SD, Debes J, Beisang D, Rattenbacher B, Louis IV, et al The hepatitis C viral nonstructural protein 5A stabilizes growth-regulatory human transcripts. Nucleic Acids Res. 2018;46(5):2537–47. 10.1093/nar/gky061 29385522 PMC5861452

[6] Guo L, Vlasova-St Louis I, Bohjanen PR. Viral manipulation of host mRNA decay. Future Virol. 2018;13(3):211–23. 10.2217/fvl-2017-0106 29750084 PMC5939598

[7] Schiff LA, Nibert ML, Tyler KL. Chapter 52: Orthoreoviruses and Their Replication In: Knipe DM, Howley PM, editors. Fields Virology, Fifth Edition Philadelphia, PA: Lippincott Williams & Wilkins; 2007 p. 1854–915.

[8] DeBiasi RL, C P, Meintzer S, Jotte R, Kleinschmidt-Demasters BK, Johnson GL, Tyler KL. Reovirus-induced alteration in expression of apoptosis and DNA repair genes with potential roles in viral pathogenesis. Journal of Virology. 2003;77(16):8934–47. 10.1128/JVI.77.16.8934-8947.2003 12885910 PMC167209

[9] O’Donnell SM, Holm GH, Pierce JM, Tian B, Watson MJ, Chari RS, et al Identification of an NF-kappaB-dependent gene network in cells infected by mammalian reovirus. J Virol. 2006;80(3):1077–86. 10.1128/JVI.80.3.1077-1086.2006 16414985 PMC1346919

[10] Smith JA, Schmechel SC, Raghavan A, Abelson M, Reilly C, Katze MG, et al Reovirus induces and benefits from an integrated cellular stress response. J Virol. 2006;80(4):2019–33. 10.1128/JVI.80.4.2019-2033.2006 16439558 PMC1367166

[11] Tyler KL, Leser JS, Phang TL, Clarke P. Gene expression in the brain during reovirus encephalitis. J Neurovirol. 2010;16(1):56–71. 10.3109/13550280903586394 20158406 PMC2891017

[12] Schmechel S, Chute M, Skinner P, Anderson R, Schiff L. Preferential translation of reovirus mRNA by a sigma3-dependent mechanism. Virology. 1997;232(1):62–73. 10.1006/viro.1997.8531 9185589

[13] Sharpe AH, Fields BN. Reovirus inhibition of cellular RNA and protein synthesis: role of the S4 gene. Virology. 1982;122(2):381–91. 6183822 10.1016/0042-6822(82)90237-9

[14] Smith JA, Schmechel SC, Williams BR, Silverman RH, Schiff LA. Involvement of the interferon-regulated antiviral proteins PKR and RNase L in reovirus-induced shutoff of cellular translation. J Virol. 2005;79(4):2240–50. 10.1128/JVI.79.4.2240-2250.2005 15681426 PMC546589

[15] Liu F. Smad3 phosphorylation by cyclin-dependent kinases. Cytokine Growth Factor Rev. 2006;17(1–2):9–17. 10.1016/j.cytogfr.2005.09.010 16289004

[16] Varga J, Pasche B. Antitransforming growth factor-beta therapy in fibrosis: recent progress and implications for systemic sclerosis. Curr Opin Rheumatol. 2008;20(6):720–8. 10.1097/BOR.0b013e32830e48e8 18946334 PMC4541793

[17] Li MO, Flavell RA. TGF-beta: a master of all T cell trades. Cell. 2008;134(3):392–404. 10.1016/j.cell.2008.07.025 18692464 PMC3677783

[18] Massague J. TGFbeta in Cancer. Cell. 2008;134(2):215–30. 10.1016/j.cell.2008.07.001 18662538 PMC3512574

[19] Massague J. TGFbeta signalling in context. Nat Rev Mol Cell Biol. 2012;13(10):616–30. 10.1038/nrm3434 22992590 PMC4027049

[20] Kubiczkova L, Sedlarikova L, Hajek R, Sevcikova S. TGF-beta—an excellent servant but a bad master. J Transl Med. 2012;10:183 10.1186/1479-5876-10-183 22943793 PMC3494542

[21] Dosanjh A. Transforming growth factor-beta expression induced by rhinovirus infection in respiratory epithelial cells. Acta Biochim Biophys Sin (Shanghai). 2006;38(12):911–4. 17151785 10.1111/j.1745-7270.2006.00234.x

[22] Mendez-Samperio P, Hernandez M, Ayala HE. Induction of transforming growth factor-beta 1 production in human cells by herpes simplex virus. J Interferon Cytokine Res. 2000;20(3):273–80. 10.1089/107999000312405 10762074

[23] Rowan AG, Fletcher JM, Ryan EJ, Moran B, Hegarty JE, O’Farrelly C, et al Hepatitis C virus-specific Th17 cells are suppressed by virus-induced TGF-beta. J Immunol. 2008;181(7):4485–94. 18802051 10.4049/jimmunol.181.7.4485

[24] Malizia AP, Keating DT, Smith SM, Walls D, Doran PP, Egan JJ. Alveolar epithelial cell injury with Epstein-Barr virus upregulates TGFbeta1 expression. Am J Physiol Lung Cell Mol Physiol. 2008;295(3):L451–60. 10.1152/ajplung.00376.2007 18621908

[25] Jiang Y, Yang M, Sun X, Chen X, Ma M, Yin X, et al IL-10(+) NK and TGF-beta(+) NK cells play negative regulatory roles in HIV infection. BMC Infect Dis. 2018;18(1):80 10.1186/s12879-018-2991-2 29439673 PMC5812185

[26] Li N, Ren A, Wang X, Fan X, Zhao Y, Gao GF, et al Influenza viral neuraminidase primes bacterial coinfection through TGF-beta-mediated expression of host cell receptors. Proc Natl Acad Sci U S A. 2015;112(1):238–43. 10.1073/pnas.1414422112 25535343 PMC4291618

[27] Denney L, Branchett W, Gregory LG, Oliver RA, Lloyd CM. Epithelial-derived TGF-beta1 acts as a pro-viral factor in the lung during influenza A infection. Mucosal Immunol. 2017. 10.1038/mi.2017.77 PMC5797694 29067998

[28] Gibbs JD, Ornoff DM, Igo HA, Zeng JY, Imani F. Cell cycle arrest by transforming growth factor beta1 enhances replication of respiratory syncytial virus in lung epithelial cells. J Virol. 2009;83(23):12424–31. 10.1128/JVI.00806-09 19759128 PMC2786720

[29] Beckham JD, Tuttle K, Tyler KL. Reovirus activates transforming growth factor beta and bone morphogenetic protein signaling pathways in the central nervous system that contribute to neuronal survival following infection. J Virol. 2009;83(10):5035–45. 10.1128/JVI.02433-08 19279118 PMC2682065

[30] Stanifer ML, Rippert A, Kazakov A, Willemsen J, Bucher D, Bender S, et al Reovirus intermediate subviral particles constitute a strategy to infect intestinal epithelial cells by exploiting TGF-beta dependent pro-survival signaling. Cell Microbiol. 2016;18(12):1831–45. 10.1111/cmi.12626 27279006

[31] Kedl R, Schmechel S., and Schiff L. Comparative sequence analysis of the reovirus S4 genes from 13 serotype 1 and serotype 3 field isolates. Journal of Virology. 1995;69:552–9. 7527088 10.1128/jvi.69.1.552-559.1995 PMC188609

[32] Jacobs BL, Ferguson RE. The Lang strain of reovirus serotype 1 and the Dearing strain of reovirus serotype 3 differ in their sensitivities to beta interferon. J Virol. 1991;65(9):5102–4. 1870214 10.1128/jvi.65.9.5102-5104.1991 PMC248978

[33] Rosen L, Hovis JF, Mastrota FM, Bell JA, Huebner RJ. Observations on a newly recognized virus (Abney) of the reovirus family. Am J Hyg. 1960;71:258–65. 14438889 10.1093/oxfordjournals.aje.a120109

[34] Furlong DB, Nibert ML, Fields BN. Sigma 1 protein of mammalian reoviruses extends from the surfaces of viral particles. J Virol. 1988;62(1):246–56. 3275434 10.1128/jvi.62.1.246-256.1988 PMC250525

[35] Raghavan A, Ogilvie RL, Reilly C, Abelson MA, Raghavan S, Vasdewani J, et al Genome-wide Analysis of mRNA Decay in Resting and Activated Primary Human T Lymphocytes. Nucleic Acids Res. 2002;30(24):5529–38. 12490721 10.1093/nar/gkf682 PMC140061

[36] Vlasova IA, McNabb J, Raghavan A, Reilly C, Williams DA, Bohjanen KA, et al Coordinate stabilization of growth-regulatory transcripts in T cell malignancies. Genomics. 2005;86(2):159–71. 10.1016/j.ygeno.2005.04.013 15979272

[37] Ibarrola N, Kratchmarova I, Nakajima D, Schiemann WP, Moustakas A, Pandey A, et al Cloning of a novel signaling molecule, AMSH-2, that potentiates transforming growth factor beta signaling. BMC Cell Biol. 2004;5:2 10.1186/1471-2121-5-2 14728725 PMC385422

[38] Kucich U, Rosenbloom JC, Abrams WR, Rosenbloom J. Transforming growth factor-beta stabilizes elastin mRNA by a pathway requiring active Smads, protein kinase C-delta, and p38. Am J Respir Cell Mol Biol. 2002;26(2):183–8. 10.1165/ajrcmb.26.2.4666 11804868

[39] Massague J. How cells read TGF-beta signals. Nat Rev Mol Cell Biol. 2000;1(3):169–78. 10.1038/35043051 11252892

[40] Sanchez-Capelo A. Dual role for TGF-beta1 in apoptosis. Cytokine Growth Factor Rev. 2005;16(1):15–34. 10.1016/j.cytogfr.2004.11.002 15733830

[41] Shi Y, Massague J. Mechanisms of TGF-beta signaling from cell membrane to the nucleus. Cell. 2003;113(6):685–700. 12809600 10.1016/s0092-8674(03)00432-x

[42] Rahimi RA, Leof EB. TGF-beta signaling: a tale of two responses. J Cell Biochem. 2007;102(3):593–608. 10.1002/jcb.21501 17729308

[43] Zhang YE. Non-Smad Signaling Pathways of the TGF-beta Family. Cold Spring Harb Perspect Biol. 2017;9(2). 10.1101/cshperspect.a022129 PMC5287080 27864313

[44] Zhang YE. Non-Smad pathways in TGF-beta signaling. Cell Res. 2009;19(1):128–39. 10.1038/cr.2008.328 19114990 PMC2635127

[45] Freudlsperger C, Bian Y, Contag Wise S, Burnett J, Coupar J, Yang X, et al TGF-beta and NF-kappaB signal pathway cross-talk is mediated through TAK1 and SMAD7 in a subset of head and neck cancers. Oncogene. 2013;32(12):1549–59. 10.1038/onc.2012.171 22641218 PMC3434281

[46] Yamashita M, Fatyol K, Jin C, Wang X, Liu Z, Zhang YE. TRAF6 mediates Smad-independent activation of JNK and p38 by TGF-beta. Mol Cell. 2008;31(6):918–24. 10.1016/j.molcel.2008.09.002 18922473 PMC2621323

[47] Derynck R, Zhang YE. Smad-dependent and Smad-independent pathways in TGF-beta family signalling. Nature. 2003;425(6958):577–84. 10.1038/nature02006 14534577

[48] Heldin CH, Landstrom M, Moustakas A. Mechanism of TGF-beta signaling to growth arrest, apoptosis, and epithelial-mesenchymal transition. Curr Opin Cell Biol. 2009;21(2):166–76. 10.1016/j.ceb.2009.01.021 19237272

[49] Syed V. TGF-beta Signaling in Cancer. J Cell Biochem. 2016;117(6):1279–87. 10.1002/jcb.25496 26774024

[50] Itoh S, Itoh F, Goumans MJ, Ten Dijke P. Signaling of transforming growth factor-beta family members through Smad proteins. Eur J Biochem. 2000;267(24):6954–67. 11106403 10.1046/j.1432-1327.2000.01828.x

[51] Moustakas A, Souchelnytskyi S, Heldin CH. Smad regulation in TGF-beta signal transduction. J Cell Sci. 2001;114(Pt 24):4359–69. 11792802 10.1242/jcs.114.24.4359

[52] Heldin CH, Moustakas A. Role of Smads in TGFbeta signaling. Cell Tissue Res. 2012;347(1):21–36. 10.1007/s00441-011-1190-x 21643690

[53] Ebisawa T, Fukuchi M, Murakami G, Chiba T, Tanaka K, Imamura T, et al Smurf1 interacts with transforming growth factor-beta type I receptor through Smad7 and induces receptor degradation. J Biol Chem. 2001;276(16):12477–80. 10.1074/jbc.C100008200 11278251

[54] Kavsak P, Rasmussen RK, Causing CG, Bonni S, Zhu H, Thomsen GH, et al Smad7 binds to Smurf2 to form an E3 ubiquitin ligase that targets the TGF beta receptor for degradation. Mol Cell. 2000;6(6):1365–75. 11163210 10.1016/s1097-2765(00)00134-9

[55] Inoue Y, Imamura T. Regulation of TGF-beta family signaling by E3 ubiquitin ligases. Cancer Sci. 2008;99(11):2107–12. 10.1111/j.1349-7006.2008.00925.x 18808420 PMC11158544

[56] Kim M, Chung YH, Johnston RN. Reovirus and tumor oncolysis. J Microbiol. 2007;45(3):187–92. 17618222

[57] Gong J, Sachdev E, Mita AC, Mita MM. Clinical development of reovirus for cancer therapy: An oncolytic virus with immune-mediated antitumor activity. World J Methodol. 2016;6(1):25–42. 10.5662/wjm.v6.i1.25 27019795 PMC4804250

[58] Ferrari G, Pintucci G, Seghezzi G, Hyman K, Galloway AC, Mignatti P. VEGF, a prosurvival factor, acts in concert with TGF-beta1 to induce endothelial cell apoptosis. Proc Natl Acad Sci U S A. 2006;103(46):17260–5. 10.1073/pnas.0605556103 17088559 PMC1859920

[59] Ferrari G, Terushkin V, Wolff MJ, Zhang X, Valacca C, Poggio P, et al TGF-beta1 induces endothelial cell apoptosis by shifting VEGF activation of p38(MAPK) from the prosurvival p38beta to proapoptotic p38alpha. Mol Cancer Res. 2012;10(5):605–14. 10.1158/1541-7786.MCR-11-0507 22522454 PMC3356490

[60] Takeshita T, Arita T, Higuchi M, Asao H, Endo K, Kuroda H, et al STAM, signal transducing adaptor molecule, is associated with Janus kinases and involved in signaling for cell growth and c-myc induction. Immunity. 1997;6(4):449–57. 9133424 10.1016/s1074-7613(00)80288-5

[61] Wang D, You Y, Lin PC, Xue L, Morris SW, Zeng H, et al Bcl10 plays a critical role in NF-kappaB activation induced by G protein-coupled receptors. Proc Natl Acad Sci U S A. 2007;104(1):145–50. 10.1073/pnas.0601894104 17179215 PMC1765424

[62] Guo B, Cheng G. Modulation of the interferon antiviral response by the TBK1/IKKi adaptor protein TANK. J Biol Chem. 2007;282(16):11817–26. 10.1074/jbc.M700017200 17327220

[63] Moser CV, Stephan H, Altenrath K, Kynast KL, Russe OQ, Olbrich K, et al TANK-binding kinase 1 (TBK1) modulates inflammatory hyperalgesia by regulating MAP kinases and NF-kappaB dependent genes. J Neuroinflammation. 2015;12:100 10.1186/s12974-015-0319-3 25997745 PMC4449530

[64] De Smaele E, Zazzeroni F, Papa S, Nguyen DU, Jin R, Jones J, et al Induction of gadd45beta by NF-kappaB downregulates pro-apoptotic JNK signalling. Nature. 2001;414(6861):308–13. 10.1038/35104560 11713530

[65] Billen LP, Shamas-Din A, Andrews DW. Bid: a Bax-like BH3 protein. Oncogene. 2008;27 Suppl 1:S93–104. 19641510 10.1038/onc.2009.47

[66] Schuman J, Chen Y, Podd A, Yu M, Liu HH, Wen R, et al A critical role of TAK1 in B-cell receptor-mediated nuclear factor kappaB activation. Blood. 2009;113(19):4566–74. 10.1182/blood-2008-08-176057 19196865 PMC2680363

[67] Weng T, Koh CG. POPX2 phosphatase regulates apoptosis through the TAK1-IKK-NF-kappaB pathway. Cell Death Dis. 2017;8(9):e3051 10.1038/cddis.2017.443 28906490 PMC5636987

[68] Yan X, Liu Z, Chen Y. Regulation of TGF-beta signaling by Smad7. Acta Biochim Biophys Sin (Shanghai). 2009;41(4):263–72. 19352540 10.1093/abbs/gmp018 PMC7110000

[69] Holm GH, Pruijssers AJ, Li L, Danthi P, Sherry B, Dermody TS. Interferon regulatory factor 3 attenuates reovirus myocarditis and contributes to viral clearance. J Virol. 2010;84(14):6900–8. 10.1128/JVI.01742-09 20463082 PMC2898218

[70] Knowlton JJ, Dermody TS, Holm GH. Apoptosis induced by mammalian reovirus is beta interferon (IFN) independent and enhanced by IFN regulatory factor 3- and NF-kappaB-dependent expression of Noxa. J Virol. 2012;86(3):1650–60. 10.1128/JVI.05924-11 22090144 PMC3264342

[71] Qin Q, Hastings C, Miller CL. Mammalian orthoreovirus particles induce and are recruited into stress granules at early times postinfection. J Virol. 2009;83(21):11090–101. 10.1128/JVI.01239-09 19710141 PMC2772771

[72] Offermann MK. Kaposi sarcoma herpesvirus-encoded interferon regulator factors. Curr Top Microbiol Immunol. 2007;312:185–209. 17089798 10.1007/978-3-540-34344-8_7

[73] Baresova P, Pitha PM, Lubyova B. Distinct roles of Kaposi’s sarcoma-associated herpesvirus-encoded viral interferon regulatory factors in inflammatory response and cancer. J Virol. 2013;87(17):9398–410. 10.1128/JVI.03315-12 23785197 PMC3754142

[74] Gupta A, Gartner JJ, Sethupathy P, Hatzigeorgiou AG, Fraser NW. Anti-apoptotic function of a microRNA encoded by the HSV-1 latency-associated transcript. Nature. 2006;442(7098):82–5. 10.1038/nature04836 16738545

[75] Nie Y, Cui D, Pan Z, Deng J, Huang Q, Wu K. HSV-1 infection suppresses TGF-beta1 and SMAD3 expression in human corneal epithelial cells. Mol Vis. 2008;14:1631–8. 18776948 PMC2529468

[76] Umbach JL, Kramer MF, Jurak I, Karnowski HW, Coen DM, Cullen BR. MicroRNAs expressed by herpes simplex virus 1 during latent infection regulate viral mRNAs. Nature. 2008;454(7205):780–3. 10.1038/nature07103 18596690 PMC2666538

[77] Meyers JM, Uberoi A, Grace M, Lambert PF, Munger K. Cutaneous HPV8 and MmuPV1 E6 Proteins Target the NOTCH and TGF-beta Tumor Suppressors to Inhibit Differentiation and Sustain Keratinocyte Proliferation. PLoS Pathog. 2017;13(1):e1006171 10.1371/journal.ppat.1006171 28107544 PMC5287491

[78] Roman A, Munger K. The papillomavirus E7 proteins. Virology. 2013;445(1–2):138–68. 10.1016/j.virol.2013.04.013 23731972 PMC3783579

[79] Arnulf B, Villemain A, Nicot C, Mordelet E, Charneau P, Kersual J, et al Human T-cell lymphotropic virus oncoprotein Tax represses TGF-beta 1 signaling in human T cells via c-Jun activation: a potential mechanism of HTLV-I leukemogenesis. Blood. 2002;100(12):4129–38. 10.1182/blood-2001-12-0372 12393612

[80] Abend JR, Imperiale MJ. Transforming growth factor-beta-mediated regulation of BK virus gene expression. Virology. 2008;378(1):6–12. 10.1016/j.virol.2008.05.009 18559281 PMC2569840

[81] Kossmann T, Morganti-Kossmann MC, Orenstein JM, Britt WJ, Wahl SM, Smith PD. Cytomegalovirus production by infected astrocytes correlates with transforming growth factor-beta release. J Infect Dis. 2003;187(4):534–41. 10.1086/373995 12599069

[82] Garneau NL, Wilusz J, Wilusz CJ. The highways and byways of mRNA decay. Nat Rev Mol Cell Biol. 2007;8(2):113–26. 10.1038/nrm2104 17245413

[83] Presnyak V, Alhusaini N, Chen YH, Martin S, Morris N, Kline N, et al Codon optimality is a major determinant of mRNA stability. Cell. 2015;160(6):1111–24. 10.1016/j.cell.2015.02.029 25768907 PMC4359748

[84] Lou CH, Dumdie J, Goetz A, Shum EY, Brafman D, Liao X, et al Nonsense-Mediated RNA Decay Influences Human Embryonic Stem Cell Fate. Stem Cell Reports. 2016;6(6):844–57. 10.1016/j.stemcr.2016.05.008 27304915 PMC4912386

[85] Chang L, Li C, Guo T, Wang H, Ma W, Yuan Y, et al The human RNA surveillance factor UPF1 regulates tumorigenesis by targeting Smad7 in hepatocellular carcinoma. Journal of experimental & clinical cancer research: CR. 2016;35:8. 26759305 10.1186/s13046-016-0286-2 PMC4711019

[86] Rehwinkel J, Letunic I, Raes J, Bork P, Izaurralde E. Nonsense-mediated mRNA decay factors act in concert to regulate common mRNA targets. RNA. 2005;11(10):1530–44. 10.1261/rna.2160905 16199763 PMC1370837

[87] Nelson JO, Moore KA, Chapin A, Hollien J, Metzstein MM. Degradation of Gadd45 mRNA by nonsense-mediated decay is essential for viability. eLife. 2016;5. 10.7554/eLife.12876 PMC4848089 26952209

[88] Bhuvanagiri M, Lewis J, Putzker K, Becker JP, Leicht S, Krijgsveld J, et al 5-azacytidine inhibits nonsense-mediated decay in a MYC-dependent fashion. EMBO Mol Med. 2014;6(12):1593–609. doi: 10.15252/emmm.201404461 25319547 10.15252/emmm.201404461 PMC4287977

