# Supplementary material for: Reovirus infection induces stabilization and up-regulation of cellular transcripts that encode regulators of TGF-β signaling
Source: PLoS One. 2018 Sep 27;13(9):e0204622. doi: 10.1371/journal.pone.0204622 (PMC6160134; doi:10.1371/journal.pone.0204622)

S1 Fig. Western blot analysis of total cell lysates from mock and reovirus-infected L929 cells. Total cellular lysates (100 µg of protein) from mock, Dearing or c87-infected L929 cells was separated by SDS-PAGE and electroblotted onto Immobilon-P transfer membranes (Millipore Corporation, Bedford, MA). Western blots were blocked in 5% dry milk in TBST buffer (50 mM Tris, 150 mM NaCL, 0.05 % Tween 20, PH 7.6) at room temperature for 2 hrs and then treated at room temperature for an additional 2 h with the following specific antibodies: rabbit polyclonal antibody against Tgif (Santa Cruz Biotechnology, Inc, Santa Cruz, CA); goat polyclonal antibody against Gdf15 (Santa Cruz Biotechnology, Inc.), and mouse polyclonal antibody against c-Myc (Calbiochem, USA). Blots were then incubated (room temperature, 2 h) with the following secondary peroxidase conjugated antibodies: donkey anti-rabbit polyclonal for Tgif (Amersham Biosciences, UK); sheep anti-mouse polyclonal for c-MYC (Amersham Biosciences) and donkey anti-goat polyclonal for Gdf15 (Santa Cruz Biotechnology, Inc.). Proteins were detected by the enhanced SuperSignal^{R} West Pico Chemiluminescent detection kit (PIERCE, Rockford, IL). The membranes were stripped using Western Blot Stripping Buffer (PIERCE, Rockford, IL) and re-probed for actin (Calbiochem, USA). Autoradiographs were quantitated by densitometric analysis using a BioRad GS-700 Imaging Densitometer (BioRad Laboratories, Hercules, CA). No increase in protein levels were observed following reovirus infection.

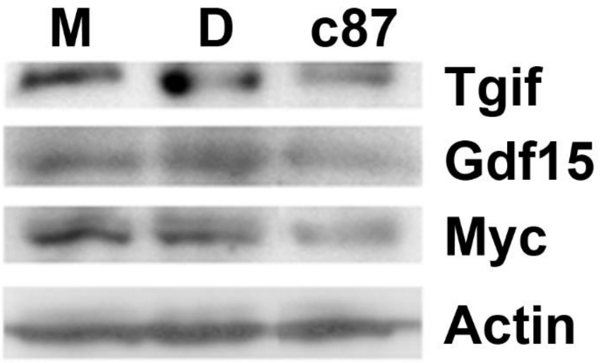

Supplement: S1 Fig — (PDF) [file pone.0204622.s004.pdf]
